# The predictive role of resilience and the ethical climate of hospital in the fatigue of surgical technologists working in operating rooms

**DOI:** 10.1186/s40359-024-01881-9

**Published:** 2024-07-08

**Authors:** Masoume Rambod, Nilofar Pasyar, Marzieh Soltanian

**Affiliations:** 1 Community Based Psychiatric Care Research Center, School of Nursing and Midwifery, Shiraz University of Medical Sciences, Shiraz, Iran; 2 Student Research Committee, Shiraz University of Medical Sciences, Shiraz, Iran

**Keywords:** Fatigue, Resilience, Climate, Ethical, Nurse, Operating room

## Abstract

**Background:**

Fatigue in surgical technologists is of paramount importance and is known as a priority because it can be regarded as a threat to the nurse’s health and patient’s safety. The fatigue level of healthcare workers can be affected by some factors, while the role of part of these factors is less known. This study aimed to determine the predictive role of resilience and the hospital ethical climate in the fatigue of surgical technologists working in operating rooms (ORs).

**Methods:**

This is a cross-sectional study conducted on 217 surgical technologists working in ORs of hospitals affiliated with Shiraz University of Medical Sciences. Data were collected using Connor-Davidson’s Resilience scale, Olson’s Hospital Ethical Climate Survey, and the Multidimensional Fatigue Inventory, and then analyzed using Pearson’s correlation coefficient and multiple regression analysis.

**Results:**

87.1% and 12.9% of surgical technologists reported low and high fatigue, respectively. All fatigue subscales had significant and negative relationships with resilience (*p* < 0.05). Moreover, the relationship between fatigue and ethical climate was significant (*p* = 0.02). The multiple linear regression model showed the predictive role of resilience in fatigue (β=-0.29, *P* < 0.001). According to the model, 10% of the change of fatigue was related to resilience and ethical climate.

**Conclusion:**

The present study demonstrated the relationship between resilience and ethical climate with fatigue. Moreover, resilience was a predictor of the surgical technologists’ fatigue, so that their fatigue decreased with increasing resilience. However, future studies are recommended to determine other factors influencing fatigue in surgical technologists.

## Introduction

The operating room (ORs) falls into the most sensitive workplaces, where the quality of work is a factor determining the quality of work at hospitals. Thus, it is expected that the highest standards are observed in ORs without even the slightest mistake. Therefore, working in such a complex environment causes job stress in ORs staff [1, 2]. It was reported that more than two-thirds of ORs personnel had occupational stress [3]. One of the consequences of this stress for ORs nurses is fatigue, which in turn affects their emotional, sensory, and cognitive abilities [4].

Fatigue is defined as a decrease in physical or mental function [5]. Occupational fatigue is a common issue among nurses [6, 7]. Researchers indicated that half of ORs nurses felt tired for months [4]. A study reported that fatigue was in a moderate level in ORs nurses [8]. Fatigue is caused by changes in central, psychological, or environmental factors [5], which increases in ORs compared to other wards due to heavy workload, high pressure, and irregular planning, followed by decreased efficiency, desire for work, and the quality of service provision [9].

The chronic fatigue effect is demonstrated by affecting the quality of life and is associated with consequences such as the reduced capacity for daily activities [10]; in fact, the employees’ fatigue negatively affects their mental health [11]. It was indicated that nurses’ occupational fatigue negatively influence their work experience, and job and colleagues’ satisfaction [6]. It led to motivation loss, disturbance in rest, and feeling of pain [4]. As such, some authors reported that the most negative effect of fatigue was on the intention to continue working [12]. A study also reports that fatigue is the major cause of errors in ORs [13]. Burnout is associated to ORs nurses’ resilience [14].

Resilience falls under the most important abilities of humans, rendering adaptation to changes, risk factors, and stressors in difficult conditions and is a factor of resistance to problems [15]. Resilience is known as the capacity of a system to adapt to challenges and the ability to turn them into opportunities and learn from challenging situations [16, 17]. It helps the nurses cope with their work environment and maintain healthy and stable psychological functioning [18]. It was reported that the improvement of resilience is an effective factor to reduce job burnout in ORs nurses [19]. Researchers also mentioned the role of resilience in increasing job satisfaction [20].

Besides resilience, other factors may be related to fatigue in surgical technologists working in ORs. Researchers have reported that occupational fatigue is associated to safety climate [6]. Occupational fatigue and occupational accidents can be reduced under the effects of underlying factors such as organizational climate [21], which is in turn influenced by the organizational ethical climate [22]. The ethical climate of the organization actually reflects the procedures and policies of the organization along with ethical consequences and is a determining factor of the accuracy and inaccuracy of the actions and ethical issues in the organization [23, 24]. The ethical climate may affect the employees’ behavior in that organization and is of paramount importance; this issue becomes more important in clinical professions that are based on commitment and ethical care [22]. Therefore, an ethical climate establishment in hospitals can provide patient care without conflict, in harmony with the patient’s rights, observing ethical sensitivities, and with ethical decisions [25].

Previous studies indicated that fatigue might compromise the safety of patients and healthcare workers in addition to the safety consequences on the performance of nurses [26, 27]. Most other studies have shown the complications caused by fatigue, the resulting problems, and the role of fatigue in the safety and functioning of surgical technologists [28]. Another study demonstrated the impact of resilience on the working conditions and stress and burnout levels in nurses [19]. The ethical climate and its relationship with nursing commitment, job burnout, and job satisfaction were also investigated in some studies [29, 30]. However, no study was found regarding the determination of fatigue-predicting factors. Due to the importance of this issue, the present study aimed to evaluate the predictive role of resilience and the hospital ethical climate in the fatigue of surgical technologists working in ORs.

## Methods

In this descriptive-analytical and cross-sectional study, data were collected from the ORs of Namazi, Shahid Faqihi, Rajaei, and Zainabieh educational centers of Shiraz University of Medical Sciences in the spring of 2022.

A sample size of 212 subjects was estimated according to “A-priori Sample Size Calculator for Multiple Regression” in “https://www.danielsoper.com/statcalc/calculator.aspx?id=1, a pilot study on 30 OR nurses, α = 0.05 (type 1 error), anticipated effect size (r^2^) of this pilot study = 0.06, desired statistical power level = 0.90, and number of predictors = 2. Then, it was raised to 222 considering a dropout of 5%. In this study, 222 questionnaires were distributed, 5 of which were incomplete and were removed, and the information of 217 OR nurses was analyzed.

The number of employees in each OR of the hospitals was determined proportional to their numbers using stratified sampling. The inclusion criteria were working as a surgical technologist, lacking underlying chronic diseases, holding at least an associate degree in surgical technology, having an experience of at least 6 months of working in ORs, and being willing to participate in the study. Exclusion criterion was incomplete filling of the questionnaires.

The study data were collected using four questionnaires, namely a demographic information questionnaire, multidimensional fatigue inventory (MFI), Connor-Davidson’s Resilience Scale (CD-RISC), and Olson’s Hospital Ethical Climate Survey (HECS).

Demographic Information Questionnaire included demographic information such as age, gender, employment status, education level, marital status, and years of employment. MFI contains 20 questions to evaluate fatigue in five dimensions, including overall fatigue, physical fatigue, mental fatigue, decreased activity, and decreased motivation. Each dimension contains four questions and the answers are scored on a 5-point Likert scale of 1 (yes, completely true) to 5 (no, completely false). Therefore, the total score of each domain and the total fatigue score are 4–20 and 20–100, respectively [31]. The total MFI cutoff was 60. Therefore, a score higher and lower than 60 indicates high and low fatigue, respectively [32]. Smets et al. (1996) first presented this questionnaire. It was used in the Dutch and Scottish samples of cancer patients undergoing radiotherapy. Good internal consistency of separate scales was obtained in both Dutch and Scottish samples with Cronbach’s alpha coefficients from 0.79 to 0.93 [31]. It was confirmed the validity and reliability of the Persian version of MFI and calculated its reliability with a Cronbach’s alpha of 0.89 [33]. The cronbach’s alpha of this study was 0.85.

Connor and Davidson (2003) prepared CD-RISC through a review of the literature in the field of resilience published in 1979–1991. This scale contains five components, namely perception of individual competence (questions 10, 11, 12, 16, 17, 23, 24, and 25), trust in individual instincts to tolerate negative emotions (questions 6, 7, 14, 15, 18, 19, and 20), positive acceptance of changes and safe relationships (questions 1, 2, 4, 5, and 8), control scale (questions 13, 21, and 22), and spiritual effects (questions 3 and 9) in 25 items. It is scored on a 5-point Likert scale ranging from 0 to 4, including never, rarely, sometimes, often, and always. In this questionnaire, the maximum and minimum scores are respectively 100 and zero, and the score of each subject is equal to the sum of the scores of each question [34]. The cut-off point of this questionnaire is 50, and scores higher than 50 indicate a more resilient person [35]. The reliability of the internal consistency coefficient of this scale was reported to be 0.89 using Cronbach’s alpha. The reliability of this scale on 24 patients with generalized anxiety disorder and post-traumatic stress disorder was reported to be 0.87. To evaluate the convergent validity of this scale, we measured its correlation using Kobasa’s hardiness questionnaire 2, which had a positive and significant correlation with this questionnaire (*r* = 0.83, *p* < 0001). A negative and significant correlation of this scale was reported with the perceived stress questionnaire (*r* = 0.76, *p* < 001) [34]. To determine the validity of this scale, Mohammadi showed the correlation coefficients of 0.41–0.64 for each score with the total score, except for item 3, and obtained its reliability with a Cronbach’s alpha coefficient of 0.89 [36]. The cronbach’s alpha of this study was 0.89.

Olson’s HECS: Developed by Olson (1998), it contains 26 components in five domains, namely colleagues (items 1, 10, 18, & 23), doctors (items 5, 9, 14, 17, 22, & 26), hospital (items 4, 8, 13, 16, 21, & 25), patients (items 2, 6, 11, & 19), and managers (items 3, 7, 12, 15, 20, & 24). According to this scale, people’s perception of the ethical climate of the hospital is measured at five levels using a Likert scale (1 = almost never, 2 = rarely, 3 = sometimes, 4 = often, and 5 = almost always). Thus, the possible minimum and maximum total scores for each person are 26 and 130, respectively. Internal consistency reliability was calculated for the total and for each of the five subscales using Cronbach’s alphas for the 26-item scale (0.91) and for the subscales of colleagues (0.73), patients (0.68), managers (0.92), hospital (0.77), and doctors (0.81). Construct validity was also evaluated with the confirmatory analysis method using statistical methods [37]. This questionnaire is scored based on the McDaniel classification. A score of ≥ 3.5 indicates the favorable employees’ opinions about the ethical climate of the hospital and suggests a positive (favorable) climate. The calculated Cronbach’s alpha also showed good reliability for the entire questionnaire (0.91) [38]. This questionnaire was translated into Persian by Mobasher et al., and its content validity was confirmed according to the review of the literature, with a favorable reliability of 0.92 [39]. The cronbach’s alpha for this study was 0.91.

The project was approved with the ethics code of IR.SUMS.NUMIMG.REC.1401.005 in Research Ethics Committees of School of Nursing and Midwifery, management and Medical Information science-Shiraz University of Medical Sciences (Approval date: 22-3-12). A written introduction letter was obtained from the Ethics Committee of Shiraz University of Medical Sciences to refer to the selected ORs of teaching-treatment centers. After coordination, permission was obtained from the head and supervisor of the department. Informed and written consent was obtained from the eligible study participants, who were assured of the anonymity of their data; then, they were invited to participate in the research.

Data were analyzed through SPSS version 27 software using descriptive (mean, standard deviation, and frequency) and inferential (Pearson’s correlation coefficient and multiple linear regression) statistics at a significance level of *P* < 0.05. In this study, Pearson’s correlation coefficients (r-value) ≥0.8, 0.4–0.8, and < 0.4 were interpreted as strong, moderate, and weak linear correlation.

## Results

The studied subjects included 217 surgical technologists consisting of 150 (69.1%) women and 67 (30.9%) men, who worked in ORs. The mean age of the participants was 33.76 (SD = 9.50) years. Moreover, married and officially employed participants comprised 61.8% and 51.6% of the participants. The participants had an average work experience of 8.61 (SD = 10.1) (Table 1).

**Table 1:** The demographic characteristics of the surgical technologists

Demographic characteristics	
Age (Mean ± SD)	33.76 ± 9.50
Gender, n (%) Male Female	67 (30.9) 150 (69.1)
Marital status, n (%) Single Married	83 (38.2) 134 (61.8)
Education, n (%) Associate degree BSc MSc	19 (8.8) 185 (85.3) 13 (6.0)
Employment status, n (%) Therapeutic-sanitary cadre Permanent Temporary-to-permanent Contractual	52 (24.4) 111 (51.6) 21 (10.1) 29 (13.8)
Years of employment (Mean ± SD)	8.61 ± 10.1

As Table 2 shows, the total mean scores of fatigue were 47.43 (SD = 9.97). Moreover, 28 (12.9%) and 189 (87.1%) participants experienced high and low fatigue levels, respectively.

**Table 2:** The mean scores of fatigue, resilience, and ethical climate in surgical technologists working in operating rooms

Variable	Min.	Max.	Mean (SD)
**Overall fatigue**	24	77	47.43 (9.97)
Dimension of fatigue			
General fatigue	5	19	11.54 (2.59)
Physical fatigue	4	19	9.32 (2.89)
Activity fatigue	4	17	8.26 (2.64)
Emotional fatigue	4	15	8.77 (2.48)
Mental exhaustion	4	15	9.52 (2.48)
** Overall resilience**	58	120	93.35 (11.62)
Dimensions of resilience			
Competency	14	40	30.05 (4.46)
Trust	14	34	24.90 (3.82)
Positive acceptance	11	25	19.58 (2.58)
Control	4	15	11.13 (2.06)
Spiritual effects	2	10	7.67 (1.78)
** Overall ethical climate** **Standardized Overall ethical climate**	9.83 1.96	24.83 4.96	18.02 (2.75) 3.56 (0.56)
Dimensions of ethical climate			
Colleagues	2	5	4.02 (0.59)
Doctors	1	5	3.32 (0.68)
Hospital	1.67	5	3.16 (0.64)
Patients	1.75	5	3.73 (0.63)
Managers	1.33	5	3.77 (0.82)

The mean overall resilience score of the participants was 93.35 (SD = 11.62). Table 2 shows the mean scores of resilience dimensions. As Table 2 shows, the mean overall score of the ethical climate was 18.02 (SD = 2.75). Moreover, standardized ethical climate was 3.56 (SD = 0.56). In this study, the colleagues and hospital domains had the highest and the lowest mean scores, respectively.

As Table 3 shows, this study indicated a statistically negative but weak association between fatigue and its subscales with resilience (*P* < 0.05). However, no significant association was observed between “spiritual effects” subscale of resilience and fatigue and its subscales. No significant association was found between “trust” as the subscale of resilience and “physical”, “activity” and “emotional” subscales of fatigue. Moreover, no significant association was reported between “competency” as subscale of resilience and “activity” subscale of fatigue. On the other hand, there was an association between the other subscales of resilience with the other subscales of fatigue (Table 3).

**Table 3:** The association between fatigue and resilience, and ethical climate and their subscales

	Fatigue	Dimensions of fatigue				
		General	Physical	Activity	Emotional	Mental
	*r*, *P*-value	*r*, *P*-value	*r*, *P*-value	*r*, *P*-value	*r*, *P*-value	*r*, *P*-value
**Overall resilience**	-0.31, < 0.001	0.64, < 0.001	0.81, < 0.001	0.76, < 0.001	0.76, < 0.001	0.77, < 0.001
*Dimensions of resilience*						
Competency	-0.28, < 0.001	-0.29, < 0.001	-0.22, 0.001	-0.13, 0.05	-0.21, 0.001	-0.23, 0.001
Trust	-0.19, 0.003	-0.26, < 0.001	-0.08, 0.20	-0.10, 0.11	-0.12, 0.07	-o.18, 0.007
Positive acceptance	-0.31, < 0.001	-0.26, < 0.001	-0.24, < 0.001	-0.20, 0.002	-0.25, < 0.001	0.20, 0.003
Control	-0.32, < 0.001	-0.24, < 0.001	-0.21, 0.002	-0.20, 0.002	-0.28, < 0.001	-0.27, < 0.001
Spiritual effects	-0.06, 0.31	0.05, 0.45	-0.002, 0.97	-0.10, 0.12	-0.17, 0.1	-0.43, 0.52
** Overall ethical climate**	-0.15, 0.02	-0.09, 0.18	-0.09, 0.16	-0.20, 0.002	-0.13, 0.05	-0.05, 0.39
*Dimensions of ethical climate*						
Colleagues	-0.26, < 0.001	-0.07, 0.27	-0.19, 0.005	-0.35, < 0.001	-0.22, 0.001	-0.16, 0.01
Doctors	-0.02, 0.76	-0.03, 0.66	-0.003,0.96	-0.03,0.64	-0.003,0.96	-0.01, 0.86
Hospital	0.02, 0.66	0.003, 0.97	0.04, 0.54	-0.10, 0.88	0.02, 0.70	0.05, 0.45
Patients	-0.21, 0.002	-0.10, 0.12	-0.12, 0.07	-0.29, < 0.001	-0.19, 0.003	-0.07, 0.26
Managers	-0.16, 0.01	-0.14, 0.03	-0.11, 0.09	-0.16, 0.01	-0.14, 0.03	-0.04, 0.53

As Table 3 shows, there was a significantly negative but weak association between fatigue and overall ethical climate (*r*=-0.15, *P* = 0.02). However, no significant association was observed between ethical climate and all dimensions of fatigue, expect for “activity” subscale. The other associations between subscales of fatigue and subscales of ethical climate are present in Table 3.

According to the multiple linear regression coefficients (Table 4), the resilience significantly predicted fatigue (β=-0.29, *P* < 0.001). According to the beta weights, resilience had a more important weight in the fatigue in surgical technologists, while the ethical climate was not a predictor of resilience (β=-0.09, *P* = 0.14). According to multiple linear regression coefficients model, 10% of the change of fatigue was related to resilience and ethical climate (*R* = 0.32, R^2^ = 0.10, Adjusted R square = 0.09).

**Table 4:** The multiple linear regression coefficients regarding the association between fatigue and resilience and ethical climate

Model	Unstandardized Coefficients		Standardized Coefficients	t	Sig.
	B	Std. Error	Beta		
(Constant)	77.39	6.16		12.55	< 0.001
Resilience	-0.25	0.05	-0.29	-4.48	< 0.001
Ethical climate	-0.35	0.23	-0.09	-1.47	0.14

## Discussion

In the present study, the mean score of fatigue in technologists was reported 47.43, and low level of fatigue was recorded in more than two-thirds of the participants. The highest and lowest mean scores belonged to general fatigue and activity, respectively. In contrast to our study, it was reported moderate to high fatigue scores in OR nurses [4]. Other studies on nurses showed that the occupational fatigue was in a moderate level [6, 7]. In ORs, heavy workload, high pressure, and irregular planning increase fatigue and concurrently endanger the employees’ job status, which can reduce efficiency, desire for work, and low service quality [9]. The experience of splashing blood or other body fluids in OR nurses, anesthetic gases, radiation, and back pain turn the OR environment into a very stressful work environment [40]. According to the present study findings, surgical technologists seem to suffer low fatigue in ORs due to the probable management of stressful conditions and possible mental and emotional peace in the OR.

In the present study, the mean score of surgical technologists’ resilience was 93.35. Similarly, a resilience score of 72 was reported in nurses [41]. A resilience score of 63.77 was reported in another study, which indicated an average level of resilience in nurses. Thus, the researchers recommended that nurses’ resilience should be improved by education and adoption of a healthy lifestyle [42]. As the mean score of resilience in our study showed, it is clearly higher than other studies conducted on nurses. Researchers indicated that resilience was negatively related to depression in nurses [43]. Therefore, resilience can be introduced as a factor that prevents or reduces the symptoms of job burnout, depression, and anxiety [44]. Yilmaz et al. believe that resilience can help the nurses cope with their work environment, maintain healthy and stable psychological functioning, and deal with the sense of depression in the clinical setting [18]. It seems that the conditions of the ORs and its complexities have increased the coping of ORs nurses in the face of continuous changes and this has made them more resilient.

This study showed standardized ethical climate was 3.56 in surgical technologists working in ORs. However, a study on rehabilitation nurses reported that the safety climate was 2.96 [45]. Moreover, a study on Iranian emergency medical services personnel reported that the mean score of ethical climate was 2.96 [46]. The means of both studies was lower than that of our study. The above studies have been conducted on nurses working on rehabilitation and emergency departments. Discipline, purposeful management with careful planning, close interactions among ORs personnel, and familiarity with physicians, staff, and ORs managers probably contributed to nurses’ better understanding of the ethical climate of the ORs.

Our study indicated that the highest and the lowest mean scores of ethical climate were reported in the colleague (M = 4.02) and hospital (M = 3.16) domains. Similarly, a study on dimensions of safety climate indicated that the dimension “relationship among nurses” had appropriate desirability [47]. Likewise, Ventovaara et al. reported a positive (M = 4) ethical climate of nurses [48]. However, a negative (M = 3.32) score of the ethical climate in surgical technologists was reported by Esmailzadeh et al. [49]. Healthcare service providers are directly affected by the ethical climate patterns adopted by hospitals, and the employees’ behavior of that organization is affected by the ethical climate [25]. Therefore, ethical climate improvement in the workplace can lead to the nurses’ satisfaction and reduce job burnout and work-induced stress [50]. Rivaz et al. showed that job burnout would decrease with the ethical climate improvement, and weakness of the ethical climate leads to job dissatisfaction, stress, job burnout, and turnover intention, along with decreased quality of care services and long-term hospitalization [51].

The results showed a significant negative but weak correlation between resilience and fatigue with a correlation coefficient of -0.31, suggesting that more resilient surgical technologists experienced less fatigue. A study on PhD and Master’s students indicated the association between fatigue and resilience [52]. Decreased resilience is associated with reduced psychosocial health and OR nurses’ adaptability to workplace stress [53]. Therefore, the development and reinforcement of personal resilience can be emphasized as a key factor in coping with a stressful work environment at hospitals [54]. It seems that surgical technologists’ views about hospital ethical climate were associated to dimensions of resilience such as competency, trust, acceptance, control, and spiritual effects.

According to the present results, a negative but weak relationship was found between the ethical climate and fatigue with a correlation coefficient of -0.15, and ethical climate improvement can weakly reduce the surgical technologists’ fatigue. In the review of the literature, no study had addressed the relationship between these two variables. Therefore, a comparison was made with similar studies. Tehrani Neshat et al. revealed that emotional exhaustion could occur in work environments with a weak ethical climate [50]. Rivaz et al. state that a favorable ethical climate reduces job burnout, and an unfavorable ethical climate leads to job dissatisfaction, stress, job burnout, and turnover intention, followed by a decrease in the quality of care services and long-term hospitalization [51].

According to the results of the regression test, resilience significantly predicts the surgical technologists’ fatigue. Organizational factors, nursing work characteristics, psychosocial factors, and individual characteristics and demands are reported to be important factors influencing the nurses’ fatigue [55]. Robert et al. asserted that mental distress caused by work pressure in health care personnel could lead to consequences, such as anxiety and depression, as well as long-term effects, such as poor resilience, fatigue, and job burnout, which could significantly influence the personnel’s health [56].

The findings of the study and the predictive impacts of resilience and the ethical climate on the fatigue of surgical technologists demonstrate the importance of a healthy work environment [57], which supports its employees, improves their performance, and ultimately facilitates better service provision to patients [58, 59]. A study indicated the correlation between the nurses’ resilience and professional ethics [60]. A healthy work environment contributes to improving nurse-doctor relationships, creating a trust-based culture, reducing medical errors, increasing job satisfaction, mitigating stress and work-induced burnout, increasing patient satisfaction, and improving treatment [61, 62]. It seems that surgical technologists’ views about positive ethical climate correlated to reduced general, physical, activity, and emotional fatigue.

## Application of findings

The present study indicates that increasing resilience and improving the ethical climate can reduce fatigue in surgical technologists. Therefore, the design and implementation of a comprehensive program, which focuses on the needs and problems of the personnel, along with attention to their living aspects, can contribute to better adaptation of technologists to working fatigue in ORs. Undoubtedly, one of the motivations of this research is applying its results to improve the quality of work in ORs. Since nurses are one of the most important and effective people in the health group, they can use the results of this research to improve the quality of care services.

One of the clinical implication of the study findings was to identify individual factors (resilience) and organizational factors (ethical climate) affecting the fatigue of nurses. Since 10% of fatigue was related to resilience and ethical climate, further studies are suggested to identify other factors correlated to fatigue.

## Limitations

A cross-sectional design and the use of self-report measurement tools were the limitations of this study.

## Conclusion

This study revealed that the level of fatigue in a vast majority of the surgical technologists was at a low level, and their resilience and ethical climate were at a satisfactory level. According to our findings, resilience is the most important predictive factor of fatigue in surgical technologists, and its increase reduces fatigue in the technologists. An association was found between the ethical climate and fatigue such that ethical climate improvement reduced fatigue in technologists. It can be concluded that increasing the resilience and ethical climate levels can effectively reduce fatigue in surgical technologists. Therefore, OR personnel, as healthcare providers on the front line, are recommended to increase their flexibility and resilience along with their activities in facing problems and move toward performance improvement and fatigue reduction through the creation and maintenance of a favorable ethical climate at hospitals.

## Data Availability

Data would be available to email of the corresponding author based on request.
